# Neural Network-Enhanced FMCW Gas Spectroscopy

**DOI:** 10.1021/acssensors.5c01825

**Published:** 2025-09-04

**Authors:** Xunzhou Xiao, Zihuai Liu, Haojia Sun, Zhen Wang, Boshi Duan, Wei Ren

**Affiliations:** † Department of Mechanical and Automation Engineering, 26451 The Chinese University of Hong Kong, Shatin, New Territories, Hong Kong SAR, 999077, China; ‡ School of Electronic Science and Engineering, 12466 Xiamen University, Xiamen 361000, China; § National Cancer Center, National Clinical Research Center for Cancer, Cancer Hospital & Shenzhen Hospital, 624688 Chinese Academy of Medical Sciences and Peking Union Medical College, Shenzhen 518116, China

**Keywords:** frequency-modulated continuous-wave, gas spectroscopy, optical gas sensing, feedforward neural network, large dynamic range

## Abstract

Detecting multicomponent gases over extensive concentration ranges with laser spectroscopy faces challenges of complex configurations, intricate spectral analysis, and reduced accuracy. Neural networks offer transformative potential for advancing laser spectroscopy by facilitating real-time optimization and automation of experimental processes. Here, we report a frequency-modulated continuous-wave (FMCW) spectroscopic system enhanced by a feedforward neural network (FNN) algorithm. FMCW encodes gas absorption spectra into optical signals, enabling both spatial localization and spectroscopic analysis. By training the FNN for the target gas components, our approach can analyze broadband superposed spectra to determine gas concentrations in the mixture. In the proof-of-concept demonstration for the measurement of C_2_H_2_ and CO_2_ mixtures, the FNN achieves high accuracy in demodulating mixed gases, with residuals less than ± 2 ppm (100–900 ppm of C_2_H_2_) and ± 0.3% (80% to 96% CO_2_), respectively. The FNN outperforms traditional concentration inversion methods regarding linear dynamic response, attaining high-precision quantification across 5 orders of magnitude (R^2^ > 0.99999). Our approach exhibits advantages of simplified signal processing and enhanced measurement accuracy, positioning it as a promising candidate for quasi-distributed sensing applications in environmental, medical, and industrial contexts.

## Graphical abstract

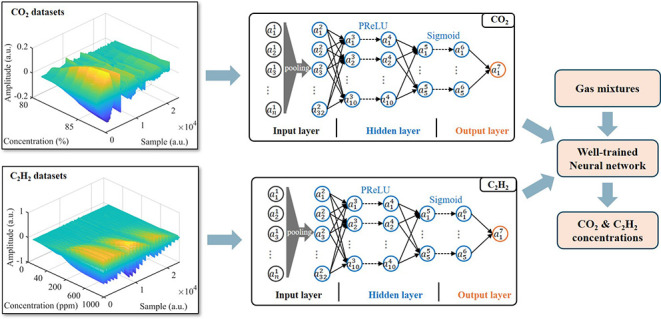

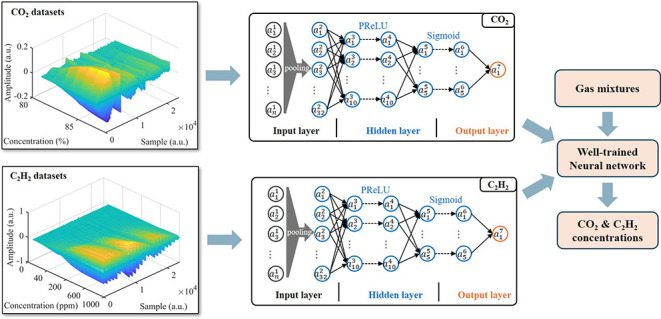

## Introduction

Multicomponent gas sensing is important across numerous applications, including the quantitative assessment of greenhouse gases for achieving carbon neutrality,^1^ tracking biomedical functions,^2^ and evaluating catalyst performance in polyethylene production.^3^ A key parameter in these contexts is the dynamic range, particularly for analyzing gas species at different atmospheric altitudes,^4^ monitoring combustion efficiency,^5^ and identifying sources of greenhouse gases.^6^ These applications often involve gas concentrations spanning more than 5 orders of magnitude. Traditional sensing methods, such as electrochemical and semiconductor gas sensors, are characterized by their low cost, fast detection, and high sensitivity.^7–10^ However, they face challenges related to cross-sensitivity and calibration issues in multicomponent environments. While gas sensor arrays can qualitatively analyze individual gases, these arrays necessitate extensive calibration due to complex cross-contamination responses.^11–13^ Additionally, techniques such as mass spectrometry and gas chromatography are inadequate for long-term, real-time monitoring and online analysis.

Laser spectroscopy is a reliable tool for multicomponent gas detection. Among its various techniques, photoacoustic spectroscopy and photothermal spectroscopy show high sensitivity and large dynamic range,^14–19^ but are susceptible to variations in light intensity and gas composition. These methods also require constant calibration and complex setups. Laser absorption spectroscopy (LAS) provides a valuable reference for achieving high-accuracy quantification with simple operation, making it the most widely used spectroscopic technique at present.^20–23^ By combining with multipass cells (MPCs) and high-finesse optical cavities, LAS enables trace gas analysis at ppt to ppq levels.^24,25^ However, extending the optical path length to enhance sensitivity narrows the dynamic range due to overabsorption, limiting their applicability to wide dynamic range sensing. Recently, Lou et al. demonstrated laser vector spectroscopy,^26^ which combines the sensitivity of absorption spectroscopy at lower gas concentrations with the linearity of dispersion spectroscopy at higher gas concentrations, extending the dynamic range to seven decades.

The overlapping absorption features of multicomponent gases necessitate the use of multiple lasers to avoid interference,^27,28^ complicating the system and increasing costs. An alternative approach involves employing a laser source with broad spectral coverage, allowing simultaneous detection of multiple gas species through single-shot measurements. This can be enhanced by aliasing spectral decoupling algorithms based on mathematical models or machine learning.^29–32^ However, traditional mathematical models, such as Pearson correlation and the least-squares method, often exhibit low inversion accuracy. Meanwhile, classical machine learning methods, including support vector machines (SVM) and k-nearest neighbors (KNN), often lose important features due to manual extraction, reducing their effectiveness. Recent advancements in deep neural networks have shown promise in analyzing complex nonlinear data, achieving high accuracy in gas classification and concentration retrieval.^33–35^ In particular, the latest neural network techniques, such as deep denoising autoencoder models, randomized smoothing, transfer learning, and spectral feature engineering, offer effective solutions in spectral analysis to enhance analysis precision across multiple applications.^36–39^ Nonetheless, challenges persist in applying these networks to LAS technology to resolve the aliasing spectral decoupling issues and the limited dynamic range.

This paper reports a broadband frequency modulated continuous wave (FMCW) spectroscopy for direct, quantitative, multispecies gas sensing. We extract the fingerprint spectral features of targeted gas molecules from mixtures through single-shot measurements. The superposed spectra are decoupled using a feedforward neural network-based analysis algorithm. We demonstrate the simultaneous detection of various levels of CO_2_ and C_2_H_2_ mixtures within the 1530 to 1540 nm range, validating our results against spectroscopic calculations with calibrated mixtures. Our approach achieves a highly linear response (R^2^ > 0.99999) and a large dynamic range for C_2_H_2_ concentrations ranging from 2 ppm to 10,000 ppm. The system fully exploits the spectral features using a wide-range tunable laser, enabling quantitative multispecies gas sensing in complex environments without the need for careful selection of noninterfering absorption lines.

### Fundamentals of FMCW Gas Spectroscopy

The proposed system for gas-phase spectroscopy combines TDLAS and FMCW to measure the transmission spectrum and reflection spectrum, respectively. TDLAS can be described by the Beer–Lambert relation:

1 $$\tau_{v}={\left(I_{t}/I_{0}\right)}_{v}=\exp\left(-\alpha_{v}L\right)$$

where $\tau_{v}$ is the transmittance, *I*_0_ and *I*_t_ are the incident and transmitted light intensity, respectively; *L* is the optical path length, α_υ_ is the absorption coefficient, which can be expressed as the following for an individual absorption transition:^40^

2 $$\alpha_{v}=S\left(T\right)P\chi_{i}\phi \left(v,T,P,\chi_{i}\right)$$

where *S­(T*) is the absorption line strength, *P* is the total pressure of the gas, and $\chi_{i}$ is the mole fraction of species *i*. By tuning the laser frequency across an absorption line, the spectrally resolved absorption feature can be precisely measured, and the gas concentration can be retrieved.

In FMCW, the returned probe light carrying absorption and dispersion spectral information is combined with the reference light to generate beat notes, which can be expressed as^41^

3 $$U\left(\omega \right)=\underset{m}{\sum }2C_{m}\sqrt{I_{P_{m}}I_{R}}H_{m}\left(\omega \right)\cos\left(\omega t_{m}\right)$$

where ω is the optical angular frequency; *Ip* and *I*_*R*_ are the light intensity of the probe and reference signal, respectively; *m* denotes the m-th reflection point in the probe arm; *t*_m_ is the time delay between the probe and reference light; and *C*_m_ is a constant that encompasses the detector responsivity, reflectivity of the reflection point, and optical loss excluding gas absorption. *H*_m_ is the transfer function of light experiencing absorption and dispersion in the gas medium, which can be expressed by^24^

4 $$H\left(\omega \right)=\exp\left(-j\left[\frac{\omega }{c}n\left(\omega \right)-j\frac{\alpha \left(\omega \right)}{2}\right]L\right)$$

where *c* is the speed of light in the vacuum. The refractive index *n* and absorption coefficient α are related by the Kramers–Kronig relation. Due to different delay times, beat notes from various reflection points have different frequencies and can be separated by performing Fourier transform (FT). By applying inverse FT to each beat term in the positive time domain, we can obtain

5 $$U_{m}\left(\omega \right)=C_{m}\sqrt{I_{P_{m}}I_{R}}H_{m}\left(\omega \right)e^{j\omega t_{m}}$$

The transmittance is given by

6 $$\tau_{m}\left(\omega \right)=\exp\left(-\alpha \left(\omega \right)L_{m}\right)={|U_{m}\left(\omega \right)|}^{2}/C_{m}^{2}I_{P_{m}}I_{R}$$

After deducting a fitted baseline, the gas concentrations can be retrieved following the same procedure as the TDLAS. Additionally, the corresponding phase change of the m-th sensing node can be extracted by

7 $$\Delta \varphi_{m}\left(\omega \right)=\arctan\left\{Im\left[U_{m}\left(\omega \right)\right]/Re\left[U_{m}\left(\omega \right)\right]\right\}-\omega t_{m}$$

where *Im* and *R*e denote taking the imaginary and real parts, respectively. The linear term ωt_m_ can be deducted by applying a linear fit. Hence, TDLAS offers superior sensitivity compared to dispersion spectroscopy, which is susceptible to laser phase noise. However, dispersion spectroscopy provides a highly linear response in optically thick media as it measures refractive index variations rather than light attenuation. By combining the TDLAS and DS, FMCW gas spectroscopy can extend the dynamic range with highly accurate quantification.^26^

### Experimental Setup

Figure 1 illustrates the schematic diagram of the proposed FMCW spectroscopic system, comprising a tunable laser, a Mach–Zehnder interferometer, a Michelson auxiliary interferometer, and a data acquisition (DAQ) system. The external-cavity diode laser (ECDL, TOPTICA) is repeatedly scanned from 1530 to 1540 nm (6535.95–6493.51 cm^–1^) at a tuning rate of 2 nm/s, with a line width of less than 10 kHz and an average output power of 10 mW. A 90:10 coupler is used for allotting the laser to the measurement interferometer’s probe arm and reference arm, respectively. A Herriott-type MPC (LaSense Technology, MPC-L), with a base length of 12.4 cm and an effective optical path length of 25 m, is placed in the probe arm of the measurement interferometer for gas analysis. The light reflected from the MPC is combined with the reference light, and their beat notes are detected by two 75-MHz balanced photodetectors (Thorlabs, PDB425C).

**Figure 1:**
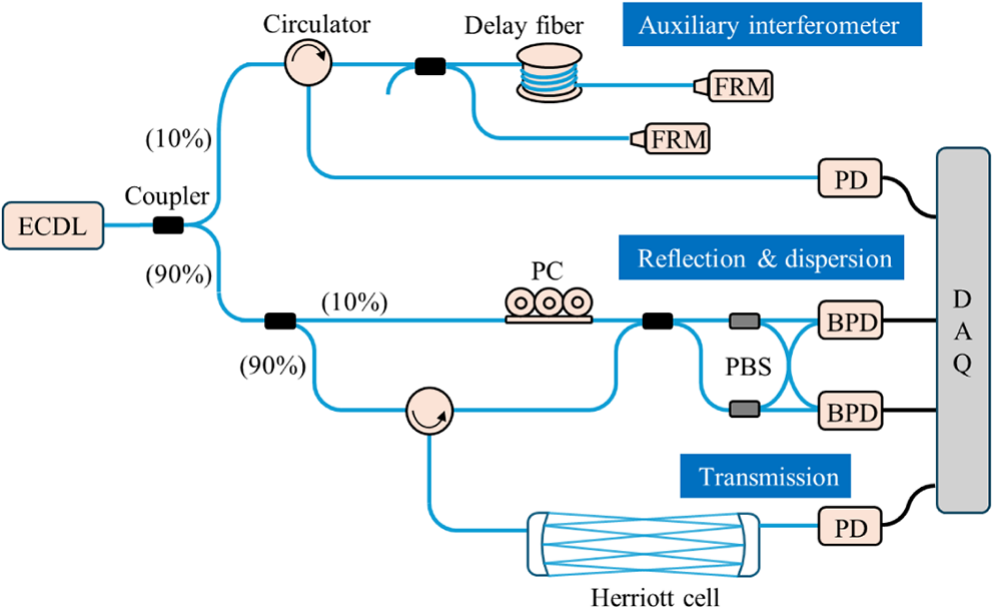
Experimental setup of FMCW gas spectroscopy. ECDL, external-cavity diode laser; PC, polarization controller; FRM, Faraday rotation mirror; PBS, polarization beam splitter; PD, photodetector; BPD, balanced photodetector; DAQ, data acquisition.

A polarization diversity reception, which consists of two polarization beam splitters (PBS) and balanced photodetectors (BPD) shown in Figure 1, is employed to reduce polarization fading in the measurement interferometer. Additionally, the auxiliary interferometer is used to compensate for the nonlinear tuning behavior of the ECDL using a phase domain interpolation resampling method.^42^ Here, two Faraday rotation mirrors (FRMs) are used to reduce the polarization fading effect, and the total optical path delay is 200 m. The beat note signals are detected by a 25-MHz photodetector (Thorlabs, PDA10D2). Finally, the transmitted light through the MPC is received by another photodetector and used for trace gas detection owing to its longer absorption path length and minimized interference noise. The output signals from the four photodetectors are recorded by a 16-bit DAQ card at a sampling rate of 10 MHz.

### Analysis of Transmission and Reflection Spectrum

The FMCW spectroscopic system operates in two modes, with the transmission spectrum for trace gas analysis and the reflection spectrum for high-concentration gas sensing. Figure 2a illustrates the transmission spectrum of 100 ppm of C_2_H_2_. By performing the phase-domain interpolation resampling, the nonlinear wavelength sweeping of the ECDL can be compensated to ensure uniform sweep rates. The transmission spectrum covers multiple absorption lines within the *v_1_+v*_2_ combination band of C_2_H_2_, which is in good agreement with the HITRAN database.

**Figure 2:**
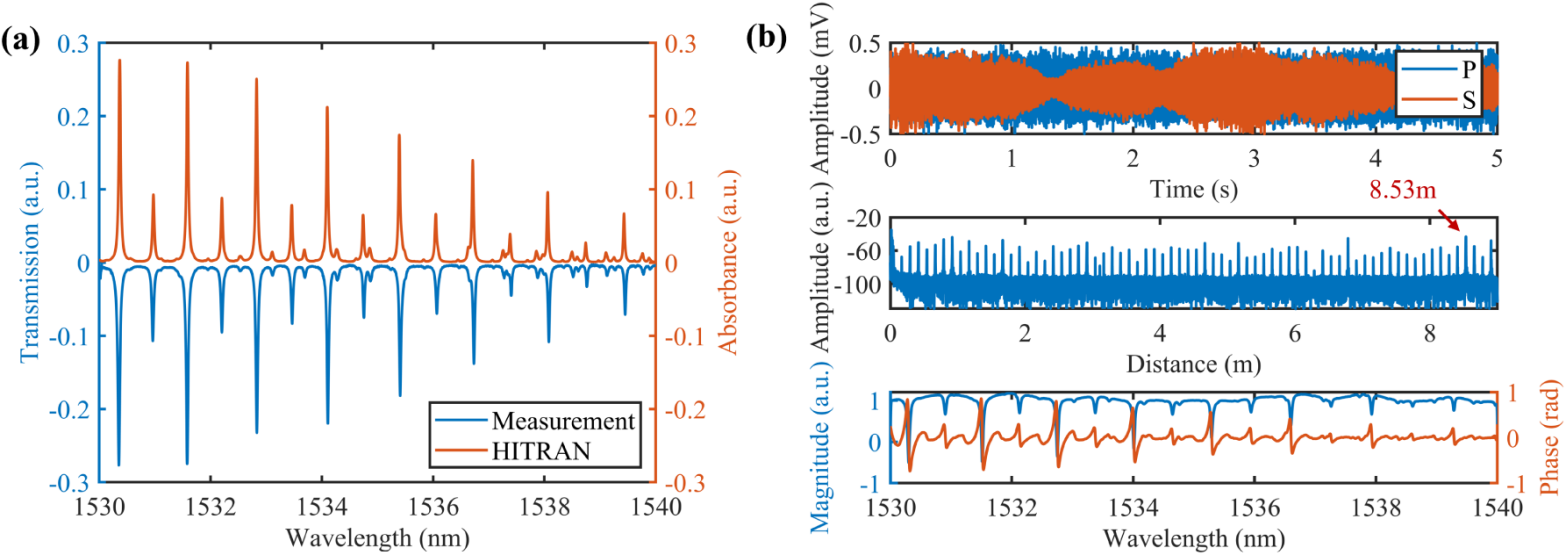
(a) Transmission spectrum of 100 ppm of C_2_H_2_ obtained using TDLAS with comparison to the HITRAN database. (b) Reflection spectrum of 1000 ppm of C_2_H_2_ obtained using FMCW. Top panel: representative beat signals from the polarization diversity reception (blue, *P* polarization; orange, *S* polarization). Middle panel: Fourier transform of the beat signal after suppressing the polarization fading. Only the data for the first 12 m are shown for clarity. Bottom panel: Retrieved absorption and dispersion spectra by applying IFT to the reflection peak for an optical length of 8.53 m.

The reflected light by the two mirrors of the Herriott cell beats with the reference light in the main interferometer. The *p*- and S-components of the detected signals are partly collected by two balanced photodetectors, as shown in Figure 2(b). The frequency-swept light from different reflection points generates beat signals with different frequencies, which can be distinguished by Fourier transform (FT). The spatial resolution, determined by SR=c/2nΔF , is estimated to be 0.12 mm at a wavelength scan range of 10 nm (△*F*), where *n* is the reflective index. The slight difference in the reflectivity at various reflection points leads to varying SNRs in the corresponding reflection and dispersion signals. We selected the reflection peak with a round-trip path length of 8.53 m by taking into account the SNR and absorption length. For demonstration purposes, this specific peak was extracted using the Gaussian window with a width of *d =* 15 cm, which is indicated in the middle panel of Figure 2b. The spectral resolution is determined to be Δs=c/2nd=1GHz , which is sufficient to resolve gas absorption spectra at atmospheric pressure. The corresponding absorption and dispersion spectra can be retrieved from the magnitude and phase of its inverse Fourier transform (IFT) results, as illustrated in the bottom panel of Figure 2b. Note that the uneven baseline in the absorption spectrum is caused by the power fluctuation during the frequency scanning. The dispersion spectrum obtained by FMCW can be used for gas sensing under very high concentrations owing to its linear response at optically thick conditions^22^.

Gas mixtures with different C_2_H_2_ concentrations were used to validate the method. All the measurements were conducted at 1 atm. Figure 3 shows the measured absorption and dispersion spectra of C_2_H_2_ from 2 ppm to 1%. As illustrated in Figure 3a,b, the optical thick condition is achieved for the higher gas concentrations, leading to the significant spectral distortion. In contrast, the dispersion signal increases linearly with the gas concentration, even under optically thick conditions, as shown in Figure 3c.

**Figure 3:**
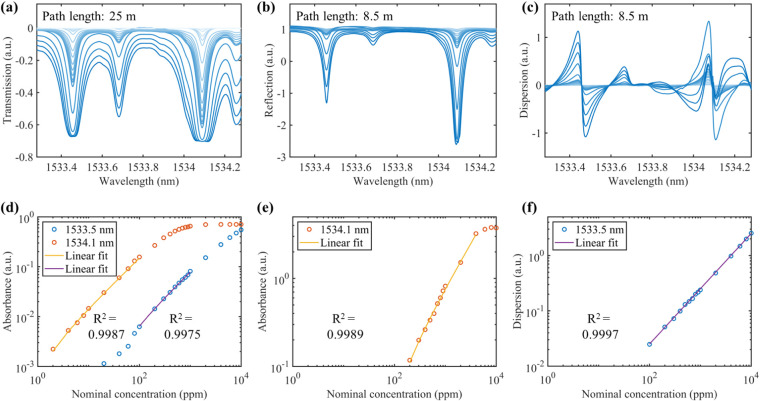
Measured (a) transmission (25 m in path length), (b) reflection (8.53 m in path length), and (c) dispersion (8.53 m in path length) spectra of C_2_H_2_ of different concentrations (2 ppm-2%). Each spectrum is obtained by 10 averages. The variation of the signal amplitude with the concentration is shown for the corresponding transmission (d), reflection (e), and dispersion (f) measurements, respectively.

One notable advantage of employing FMCW for gas detection is the inverse relationship between the laser wavelength sweep range and spatial resolution.^32^ This allows the high spatial resolution (tens of micrometers) while covering a broad spectral range (tens of nanometers). Although FMCW can extend the dynamic range, this benefit comes at the cost of careful selection of absorption lines and optical paths. As illustrated in Figure 3d, in transmission mode, the absorption line at 1534.1 nm exhibits a linear response from 2 to 100 ppm at an optical path length of 25 m, while the absorption line at 1533.5 nm responds linearly from 100 to 1000 ppm. In reflection mode, the line at 1534.1 nm shows linearity from 200 to 1000 ppm for an 8.53 m path length, as shown in Figure 3e. In dispersion mode, the line at 1533.5 nm presents linearity across a range from 100 to 10,000 ppm, as shown in Figure 3f. Hence, achieving linearity across a wide dynamic range remains a complex task. Additionally, the traditional concentration inversion method is ineffective for multicomponent gases due to interference from overlapping spectra. Accurately identifying individual gas components and their concentrations in overlapping spectra remains a critical issue that needs to be addressed.

## FNN-Based Multigas Spectral Analysis

To address these challenges, we propose a novel spectral analysis algorithm for FMCW using a feedforward neural network (FNN). While FNNs show inherent limitations in hierarchical feature extraction and modeling long-range dependencies compared to sophisticated neural architectures such as CNNs and Transformers,^36,37^ FNNs offer distinct advantages in architectural simplicity and low resource consumption for equivalent functional performance, making them ideal for edge computing. Guided by hardware constraints and real-time processing needs, we adopted FNNs as the network framework, balancing deployability with core functionality. In this study, C_2_H_2_ and CO_2_ are selected as the target gases due to their significant spectral interference within the 1530–1540 nm range, as shown in Figure 4, based on the HITRAN database. We designed two specialized neural network models for these two gases, sharing a unified architectural framework with different configurations of their input and output layers. By training the networks for each specific gas, we can effectively predict the concentration of their mixtures. This architecture boasts high scalability and requires only a few samples.

**Figure 4:**
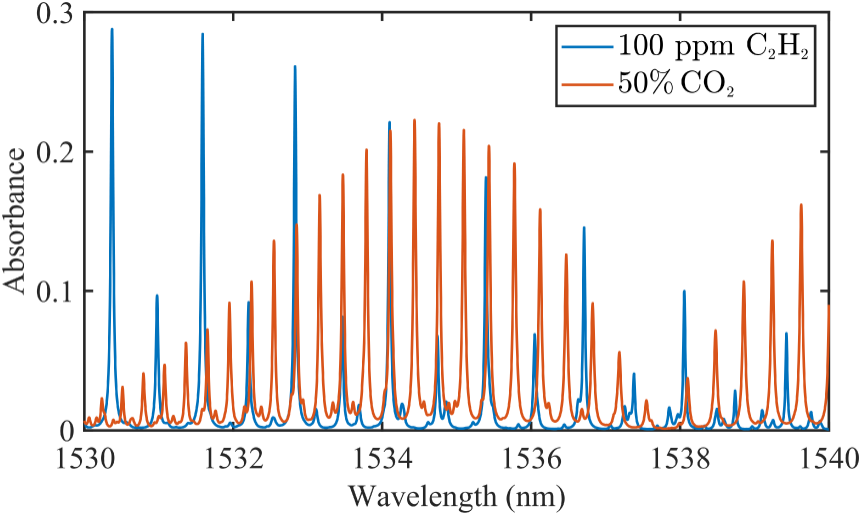
HITRAN simulation of C_2_H_2_ and CO_2_ spectra (1530–1540 nm) for a path length of 24 m.

We conducted 100 measurements for each gas concentration, with C_2_H_2_ spanning from 2 ppm to 10,000 ppm at logarithmical intervals and CO_2_ from 10% to 100% at linearly equal intervals. The transmission, reflection, and dispersion spectra for each concentration were calculated and compiled into a data set. We randomly selected 80 measurements for training, 10 for validation, and reserved 10 for inference, ensuring the test set was exclusively used for model evaluation. To mitigate the influence of baseline drift during the data collection and ensure a smooth transition among three segments, a 50-point interval differencing operation was applied to the transmission and reflection spectra, as illustrated in Figure 5. The differencing process, a simplified spectral feature engineering proposed by Sy et al.,^38^ can suppress flat regions of the absorption line shape and compresses the dynamic range of absorbance values, thereby enhancing the visual discrimination of subtle spectral features. Figure 3 indicates that integrating measurement data with 25 and 8.53 m optical paths, along with their respective absorption peaks, enables comprehensive coverage of the entire concentration range. Hence, our training data set selectively incorporates these sets of data, including transmission spectra with 25 m path length, and reflection and dispersion spectra with 8.53 m path length, to optimize neural network performance for accurately predicting gas concentrations across a broad dynamic range. The differential operation also prevents substantial discrepancies at the junctions of fused data segments, ensuring consistent feature extraction during neural network training. Hence, the per-segment normalization process is not required.

**Figure 5:**
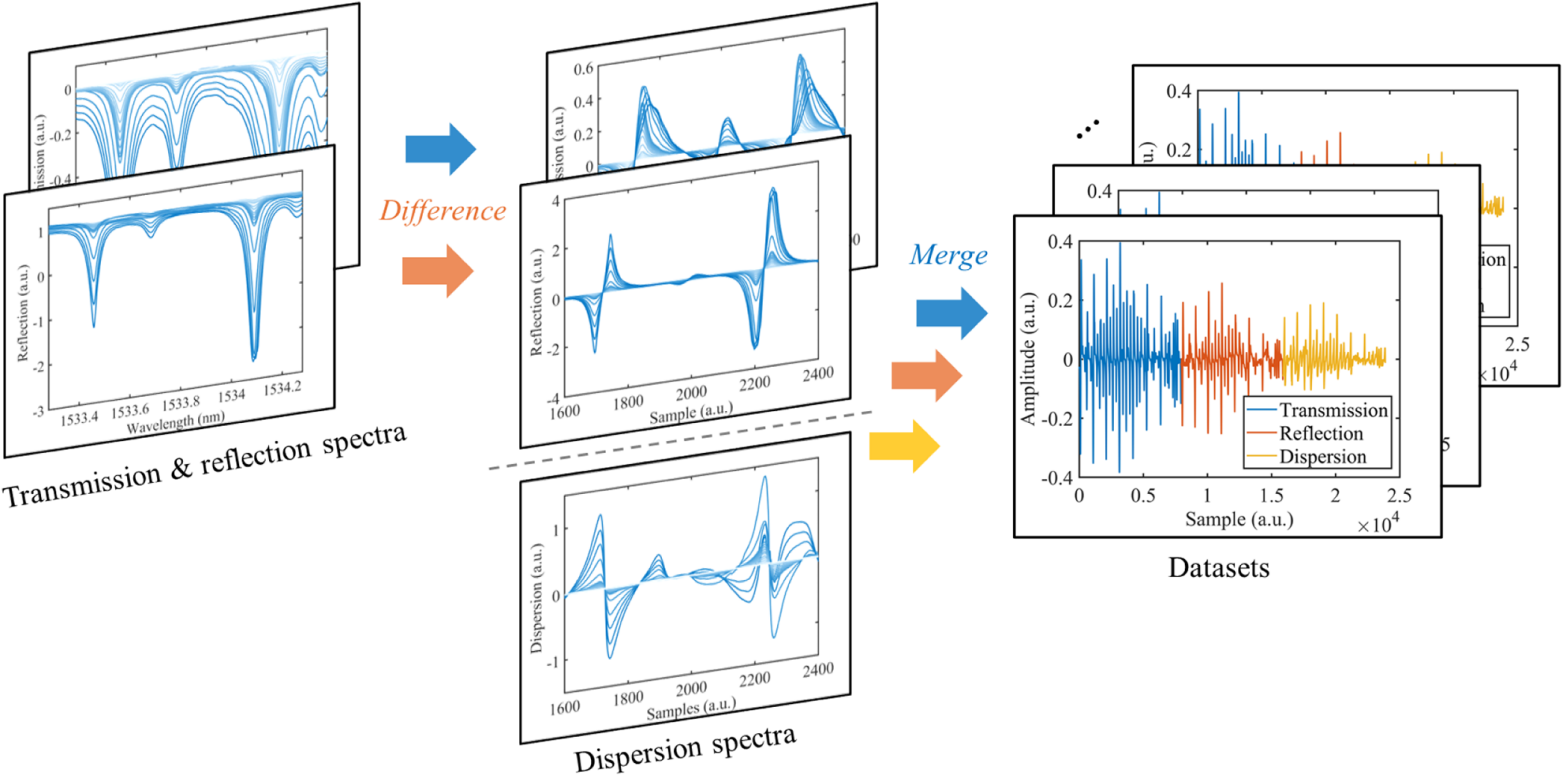
Data preprocessing for FMCW spectroscopy. The differential process is used to mitigate the baseline drift within each data set and offset discrepancies among the three segments.

The data sets utilized for training C_2_H_2_ and CO_2_ neural networks contain 2000 and 1200 samples, respectively. Figure 6 shows the structure of the FNN, which includes an input layer, a hidden layer, and an output layer. The input layer has 24,572 neurons, corresponding to the length of the fused spectra, while the output layer has 1 neuron for the predicted gas concentration. The hidden layer comprises three levels, with 32, 10, and 5 neurons, respectively. The input layer is connected to the hidden layer via average pooling. The learning rate is set at 0.01, and the mean squared error (MSE) serves as the loss function. All training procedures were conducted on an NVIDIA RTX4090 GPU. To mitigate the impact of high concentrations on the MSE of low concentrations, labels were logarithmically transformed before training. The main performance metrics for each training iteration are also shown in Figure 6. The MSE of the training and validation data decreases with increasing iterations, achieving the optimal performance after 842,359 and 88,861 iterations, respectively. Considering that CO_2_ concentrations are 3 orders of magnitude higher than C_2_H_2_, the training loss for CO_2_ (100 ppm) is higher than that for C_2_H_2_ (1 ppm). The model’s robust performance on the test set supports its application in practical measurements. The trained neural network is then deployed in FMCW spectroscopy for multicomponent gas measurements.

**Figure 6:**
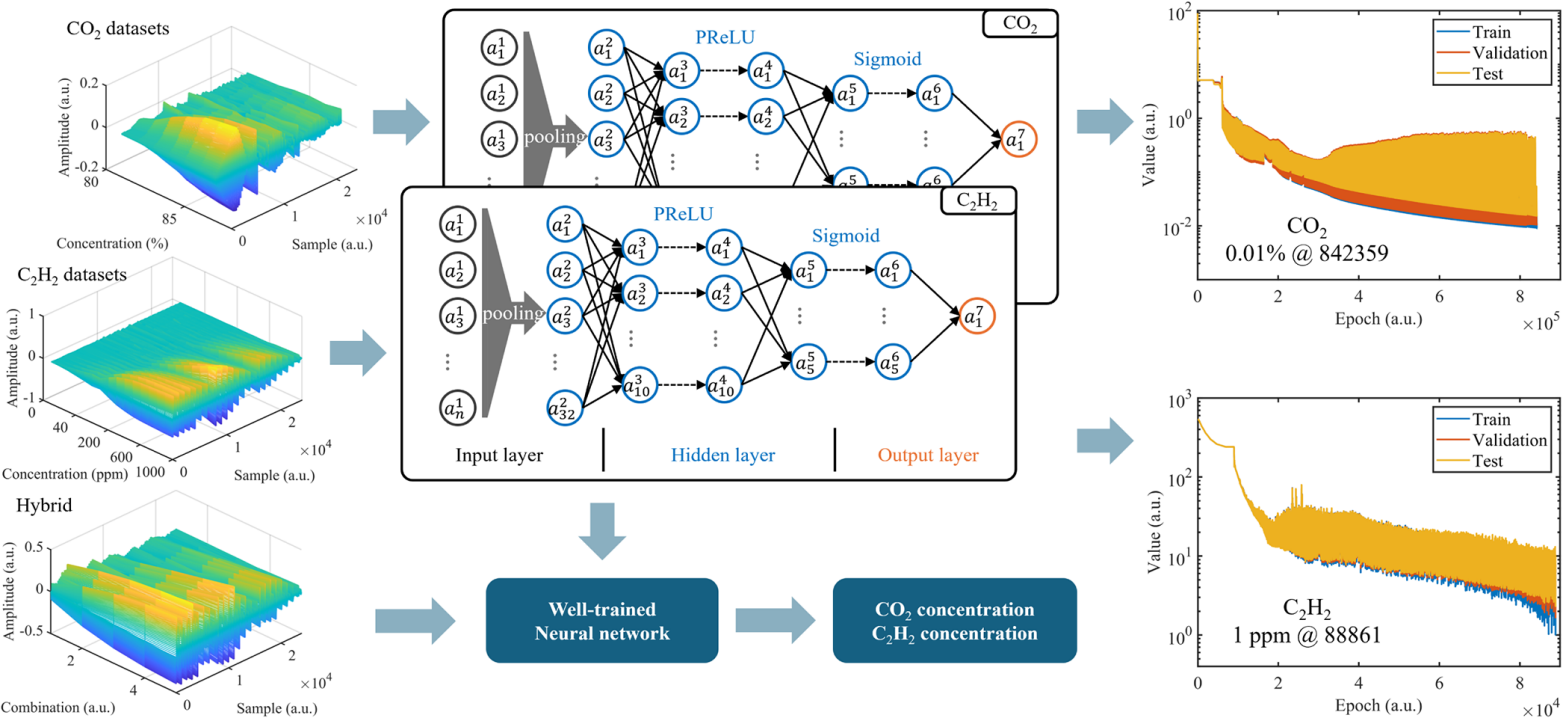
Construction of FNN-based spectral analysis algorithm and the evolution of the cost function during training for finding the concentration of C_2_H_2_ and CO_2_.

To assess the performance of the proposed FNN algorithm, diluted C_2_H_2_ (100–1000 ppm) and CO_2_ (80% – 100%) mixtures were introduced into the MPC. The predicted concentrations of C_2_H_2_ and CO_2_ via FNN are compared with those calculated using spectral fitting, with partial results shown in Figure 7. The root mean squared error (RMSE) for C_2_H_2_ and CO_2_ using the FNN is 2 ppm and 0.087%, respectively. In contrast, the RMSE for the calculated C_2_H_2_ and CO_2_ concentrations is 5.97 ppm and 0.31%, respectively. The residuals of the FNN-predicted concentrations are significantly lower than for calculated values, attributed to the high SNR across transmission, reflection, and dispersion spectra.

**Figure 7:**
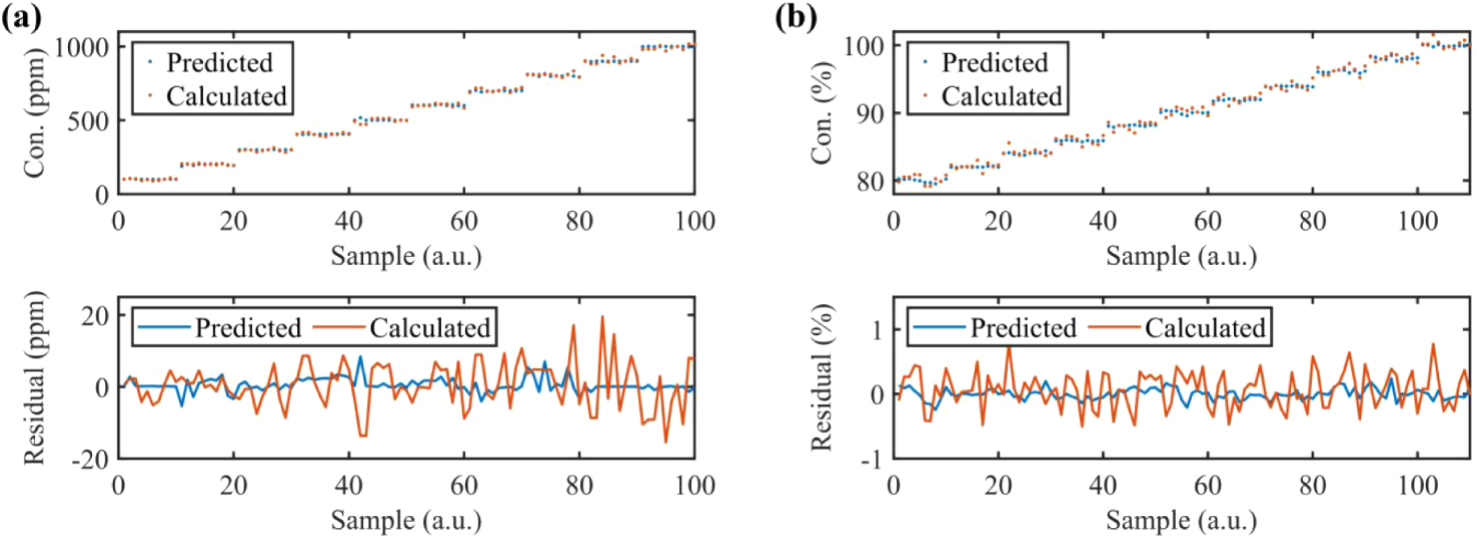
Concentration estimation for (a) C_2_H_2_ and (b) CO_2_. The top panel is magnified twice for clarity; the bottom panel is the corresponding residual of the predicted and calculated concentrations.

To assess the antialiasing capability, five gas mixtures containing both CO_2_ and C_2_H_2_ were prepared for analysis. Figure 8a illustrates the significant aliasing interference in the absorption spectra of these mixtures, complicating concentration inversion. The concentration gradient diagram, in Figure 8b, shows the predicted C_2_H_2_ and CO_2_ concentrations using the neural network. The predicted concentrations of the two gases align with the preset values and are not affected by aliasing absorption spectra. The residuals for C_2_H_2_ and CO_2_ are within ± 2 ppm and ± 0.3% in a range of 100 ppm to 900 ppm and 80% to 96%, respectively, as shown in Figure 8c. Note that signal fluctuations at the same concentration level mainly stem from an unstable gas supply in the dilution system. This demonstrates that the FNN algorithm provides stable predictions despite aliasing interference between gases. In contrast, traditional spectral fitting methods often experience significant accuracy degradation under similar conditions.

**Figure 8:**
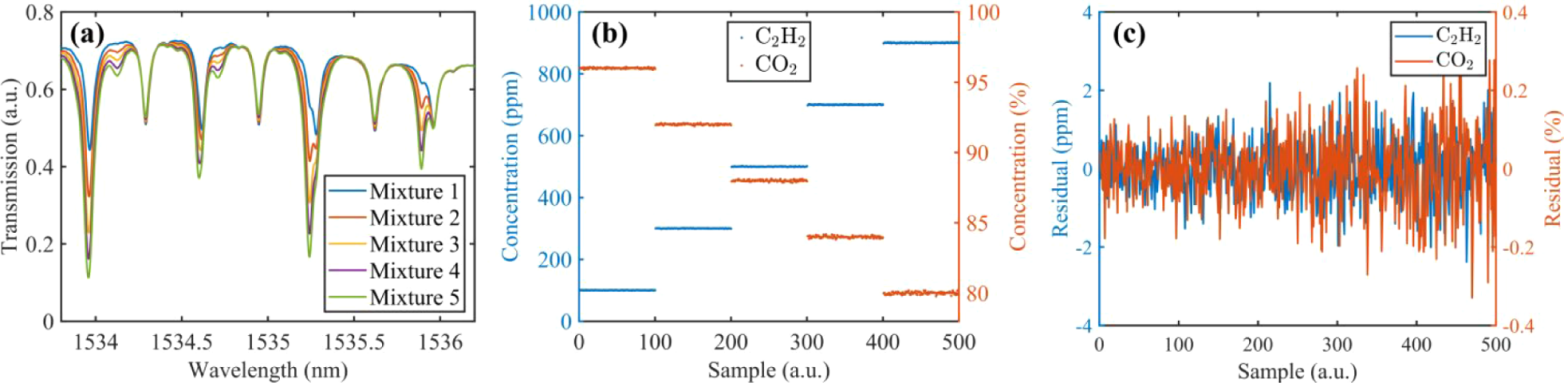
(a) Measured aliasing spectra of C_2_H_2_ and CO_2_ at various concentrations. Mixture 1:100 ppm of C_2_H_2_ and 96% CO_2_; mixture 2:300 ppm of C_2_H_2_ and 92% CO_2_; mixture 3:500 ppm of C_2_H_2_ and 88% CO_2_; mixture 4:700 ppm of C_2_H_2_ and 84% CO_2_; mixture 5:900 ppm of C_2_H_2_ and 80% CO_2_. (b) Predicted C_2_H_2_ and CO_2_ concentrations in the five mixtures. (c) Residual of the predicted concentrations.

Additionally, we conducted gas measurements of C_2_H_2_ spanning a wide concentration range of 2–10,000 ppm. Figure 9 illustrates the concentration measurements determined by both the spectral fitting method and the FNN method, with error bars magnified 20 times for clarity. In the spectral fitting process, different spectra, optical path lengths, and absorption lines are selected based on concentration levels. For low concentrations (<100 ppm), absorption spectra with higher SNR are adopted; for high concentrations (>1,000 ppm), reflection and dispersion spectra with an optical path of 0.97 m are used due to their superior linearity; for intermediate concentrations (100–1000), both transmission and reflection spectra with an optical path of 8.53 m are employed to enhance accuracy and precision. Following data fusion, the linear fit achieves an R^2^ value of 0.99976, though biases and significant errors exist at low concentrations due to low SNR. In contrast, the FNN-based analysis achieves the RMSE of 4.02 ppm and an R^2^ of more than 0.99999 without noticeable bias, indicating a nearly perfect linear response over the entire concentration range. This approach simplifies processing by eliminating the need for selecting different spectra for varying gas concentrations, enhancing measurement accuracy.

**Figure 9:**
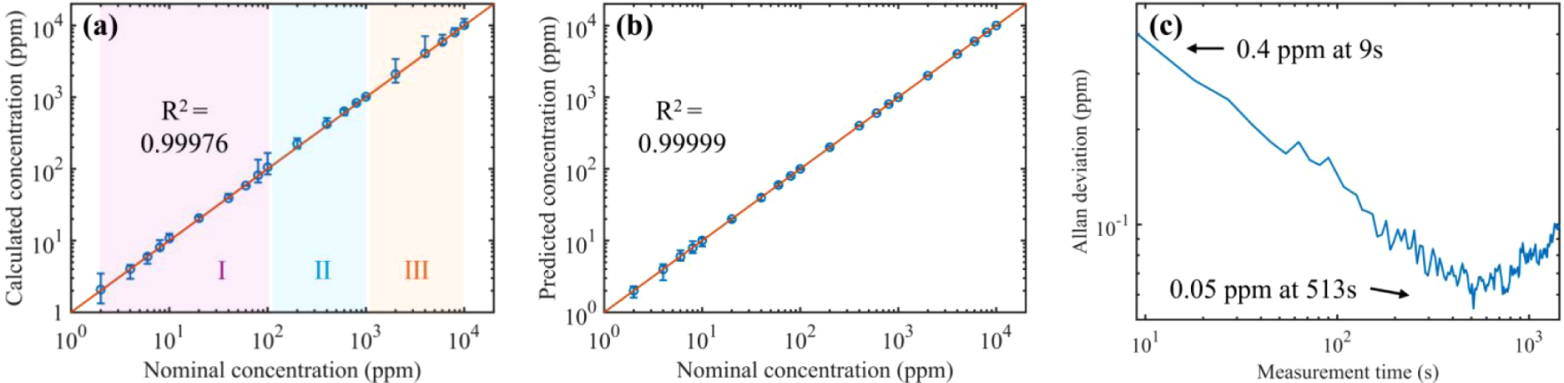
Comparison of (a) calculated and (b) predicted C_2_H_2_ concentrations with the nominal concentrations. Region I represents using transmission spectra only to calculate the concentration, region II represents incorporating both transmission and reflection to calculate the concentration, and region III represents incorporating both reflection and dispersion spectra to calculate the concentration. Error bars represent the standard deviation of 20 measurements (magnified 20 times for clarity). (c) Allan-Werle deviation plot yielded by continuous measurement of 10 ppm of C_2_H_2_ over 2 h.

To evaluate the minimum detection concentration (MDC), Allan-Werle variance analysis was performed on continuous measurements of 10 ppm of C_2_H_2_ over 2 h. As shown in Figure 9c, the MDC of the system is 0.05 ppm at the averaging time of 513 s. The noise in the Allan variance is primarily caused by fluctuations in laser power. The sensing dynamic range is defined by the lower detection limit (LDL) and the upper detection limit (UDL). Taking the MDC of 0.05 ppm as the LDL and the measured highest concentration of 1% as the UDL, the experimentally realized dynamic range of the current laser spectroscopic system is 10.^5^ This range can be further extended by choosing an MPC with a longer optical path.

## Conclusion

We demonstrated a novel multicomponent gas sensing approach using FMCW gas spectroscopy enhanced by a neural network algorithm. The proposed FNN-based gas analysis algorithm integrates various absorption lines, optical path lengths, and spectral methods to enable quantitative analysis of multicomponent gases across a wide dynamic range. Our method achieved a dynamic range of five decades in C_2_H_2_ measurement with an RMSE of 4.02 ppm and a linearity of 0.9999. By extending the wavelength scanning range of the FMCW, we achieved simultaneous C_2_H_2_ and CO_2_ mixture measurements, and the residuals of C_2_H_2_ and CO_2_ in mixed gases sensing are less than ± 2 ppm and 0.3% in the range of 100–1000 ppm and 80–100%, respectively. The FNN-based spectral analysis provides simple processing, synchronous measurement, and robust anti-interference capabilities. Notably, our approach shows significant potential for assessing environmental pollution and monitoring industrial emissions, particularly in scenarios where real-time estimation of components and concentration poses challenges for calibration. In the future, we will consider pressure-induced spectral changes through augmentations to make it more suitable for practical application scenarios.
